# Morphometric Optic Nerve Head Analysis in Glaucoma Patients: A Comparison between the Simultaneous Nonmydriatic Stereoscopic Fundus Camera (Kowa Nonmyd WX3D) and the Heidelberg Scanning Laser Ophthalmoscope (HRT III)

**DOI:** 10.1155/2016/4764857

**Published:** 2016-05-30

**Authors:** Siegfried Mariacher, Stephanie Hipp, Robert Wirthky, Gunnar Blumenstock, Karl-Ulrich Bartz-Schmidt, Focke Ziemssen, Ulrich Schiefer, Bogomil Voykov, Kai Januschowski

**Affiliations:** ^1^Knappschaft Eye Clinic, Knappschaft Hospital Saar GmbH, 66280 Sulzbach, Germany; ^2^Ophthalmology Clinic, Katharinenhospital, Klinikum Stuttgart, 70174 Stuttgart, Germany; ^3^University Eye Clinic Tuebingen, Centre for Ophthalmology, Eberhard Karls University of Tuebingen, 72076 Tuebingen, Germany; ^4^Department of Clinical Epidemiology and Applied Biostatistics, Eberhard Karls University of Tuebingen, 72076 Tuebingen, Germany; ^5^Institute for Ophthalmological Research, Eberhard Karls University of Tuebingen, 72076 Tuebingen, Germany; ^6^Competence Center “Vision Research”, Aalen University of Applied Sciences, 73430 Aalen, Germany

## Abstract

*Purpose.* To investigate the agreement between morphometric optic nerve head parameters assessed with the confocal laser ophthalmoscope HRT III and the stereoscopic fundus camera Kowa nonmyd WX3D retrospectively.* Methods.* Morphometric optic nerve head parameters of 40 eyes of 40 patients with primary open angle glaucoma were analyzed regarding their vertical cup-to-disc-ratio (CDR). Vertical CDR, disc area, cup volume, rim volume, and maximum cup depth were assessed with both devices by one examiner. Mean bias and limits of agreement (95% CI) were obtained using scatter plots and Bland-Altman analysis.* Results.* Overall vertical CDR comparison between HRT III and Kowa nonmyd WX3D measurements showed a mean difference (limits of agreement) of −0.06 (−0.36 to 0.24). For the CDR < 0.5 group (*n* = 24) mean difference in vertical CDR was −0.14 (−0.34 to 0.06) and for the CDR ≥ 0.5 group (*n* = 16) 0.06 (−0.21 to 0.34).* Conclusion.* This study showed a good agreement between Kowa nonmyd WX3D and HRT III with regard to widely used optic nerve head parameters in patients with glaucomatous optic neuropathy. However, data from Kowa nonmyd WX3D exhibited the tendency to measure larger CDR values than HRT III in the group with CDR < 0.5 group and lower CDR values in the group with CDR ≥ 0.5.

## 1. Introduction

Glaucoma is one of the leading causes for irreversible blindness in western nations [1]. Morphometric optic nerve head parameters like cup-to-disc-ratio (CDR), cup and rim volume, and mean or maximum cup depth are surrogates for glaucomatous optic neuropathy. These parameters are significantly correlated with visual field indices such as mean deviation (MD) and pattern standard deviation (PSD) [2, 3]. While morphometric optic nerve head analysis is useful for detecting early structural changes [4], perimetry is used for monitoring functional changes [5]. The above-mentioned approaches are complementary [6]. Morphometric examinations are an objective way to monitor both manifestation and progression of optic neuropathy by detecting changes in optic nerve head parameters in patients with primary open angle glaucoma and ocular hypertension [7, 8].

Evaluation of the optic nerve head with the historically used two-dimensional planimetry was particularly difficult in patients with extremely small or large discs, regarding detection of glaucomatous damage in small discs and progression in large discs [7]. In 1988 the confocal laser ophthalmoscope Heidelberg Retina Tomograph (HRT) was introduced. A laser diode with a wavelength of 670 nm was used for scanning purposes [4, 9, 10]. Currently the HRT has become one of the standard tools for three-dimensional topographic analysis of the optic nerve head [11, 12]. The Kowa nonmyd WX3D is a stereometric fundus camera that is able to take two photographs simultaneously [13, 14]. Thus a highly reproducible (in contrast to sequential shift of a single camera) and most importantly real (in contrast to HRT) stereoscopic photo is acquired and changes can be analyzed qualitatively via anatomic examination as well as quantitatively via stereometric optic nerve head parameters [15, 16], per pixel diversion. If the analysis of optic nerve head parameters with Kowa nonmyd WX3D and HRT measurements would be comparable, the virtues of stereoscopic photography and optic nerve head morphometric analysis could be combined. This study was conducted to investigate the agreement between HRT III and Kowa nonmyd WX3D in patients with primary open angle glaucoma.

## 2. Materials and Methods

40 eyes of 40 patients with primary open angle glaucoma were monitored at the glaucoma outpatient ward at the University Eye Hospital Tuebingen and analyzed retrospectively representing a rough average of the possible eligible patient population. Patients with chronic, progressive optic neuropathies with characteristic morphological changes at the optic nerve head and retinal nerve fiber layer in the absence of other ocular disease or congenital anomalies were defined as having an open angle glaucoma. Progressive retinal ganglion cells death and visual field loss are associated with these changes and were mandatory to be included into this analysis. Eligible patients were divided into 2 groups regarding their vertical CDR assessed with HRT III (Heidelberg Retina Tomograph III, Heidelberg Engineering GmbH, Heidelberg, Germany). Patients with a vertical CDR lower than 0.5 formed one group and patients with a vertical CDR higher than or equal to 0.5 formed the second group. Exclusion criteria were media opacities (cornea, lens, and vitreous), advanced ametropia (spherical refraction exceeding 4 diopters or astigmatism exceeding 2 diopters), ophthalmological diseases affecting the optic nerve head other than glaucoma, or impaired quality indices with regard to HRT (namely, topography standard deviation (TSD) > 30 or intertest variation of reference height > 25 *μ*m). Patients who underwent refractive or vitreoretinal surgery were also excluded from the analysis. Corneal pachymetry measurements were included into the analysis for correction of the HRT III results.

The contour line of the disc needs to be placed automatically or manually on the top of the disc margin and serves as a reference for follow-up examinations. The cup contour will be reassessed within each subsequent session [12]. The cup contour line alignment was manually performed in both Kowa nonmyd WX3D and HRT III by one examiner. Vertical CDR, disc area, cup volume, rim volume, and maximal cup depth were analyzed with both methods. Agreement between HRT III and Kowa nonmyd WX3D (Kowa Company Ltd., Nagoya, Japan) was analyzed using scatter plots and Bland-Altman analysis [17]. Mean bias and upper and lower limits of agreement (95% confidence interval (CI)) were evaluated. All statistical analyses were performed using IBM SPSS Statistics 22.0 for Windows (IBM Corp., Armonk, NY, US).

The current investigation was approved by the ethics committee of the University of Tuebingen (Germany). This study was performed in accordance with the ethical tenets outlined in the Declaration of Helsinki.

## 3. Results

The study included 40 eyes from 40 patients with primary open angle glaucoma. Twenty-four eyes with a CDR < 0.5 and 16 eyes with a CDR ≥ 0.5 were analyzed. 27 female patients (67.5%) and 13 male patients (32.5%) were included in this survey. In the group with CDR < 0.5 18 female (75%) and 6 male (25%) patients and in the group with CDR ≥ 0.5 9 female (56%) and 7 male (44%) patients were investigated.

**Figure 1:**
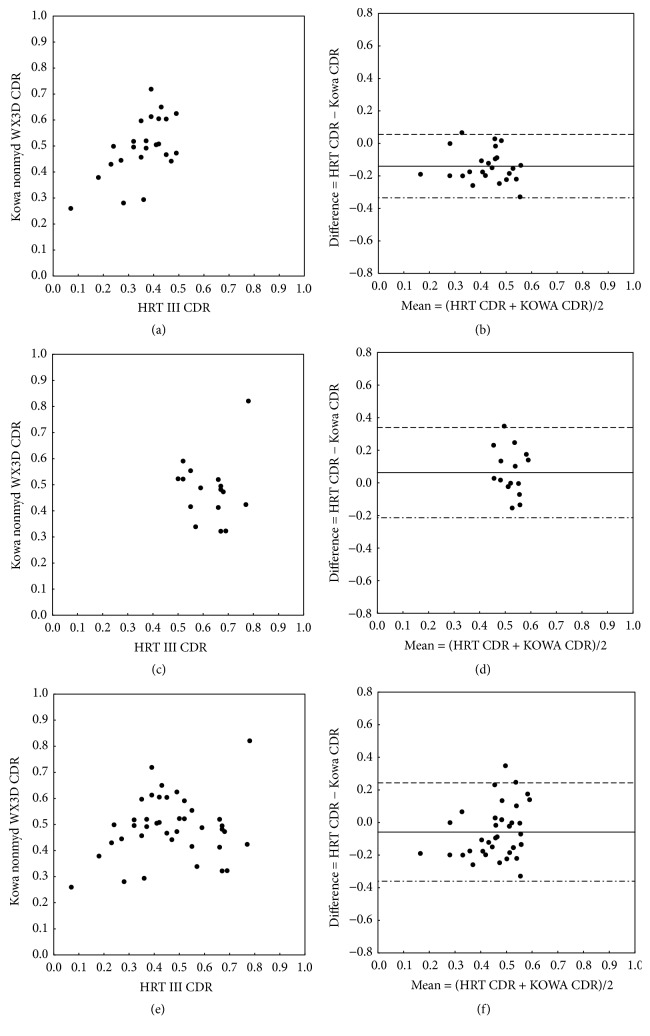
Scatter plots (a, c, e) and Bland-Altman plots (b, d, f) showing the correlation between (vertical) CDR measurements using HRT III versus Kowa nonmyd WX3D, separated for the group with CDR < 0.5 (a, b) and the group with CDR ≥ 0.5 (c, d). Overall comparison is visualized in plots (e, f). In Bland-Altman plots solid lines indicate mean differences between CDR measured with HRT III and Kowa nonmyd WX3D. The 95% limits of agreement are indicated by the upper dashed lines and the lower dot-dashed lines.

The agreement between morphometric optic nerve head parameters assessed with HRT III and Kowa nonmyd WX3D was analyzed using mean differences and limits of agreement (95% CI). Bland-Altman plots and scatter plots were generated to compare and validate measurements from both devices (Figure 1).  Table 1 shows the mean CDR, cup volume, rim volume, and maximum cup depth measured with HRT III and Kowa nonmyd WX3D. The mean differences with corresponding limits of agreement between HRT III measurements and Kowa nonmyd WX3D measurements are shown in Table 2.

Overall analysis of vertical CDR showed a slight negative mean difference between HRT III and Kowa nonmyd WX3D measurement (−0.06; limits of agreement −0.36 to 0.24). Mean disc area and rim volume were larger, but cup volume and maximal cup depth were smaller in Kowa nonmyd WX3D assessment in comparison to the HRT III assessment (Table 2).

Scatter plots and Bland-Altman plots (Figure 1) also showed a negative mean difference (−0.14; limits of agreement −0.34 to 0.06) between vertical CDR assessed with HRT III and Kowa nonmyd WX3D in the CDR < 0.5 group, indicating that the Kowa nonmyd WX3D was measuring larger CDR values than the HRT III in this group.

Cup volume and maximum cup depth showed a positive mean difference between HRT III and Kowa nonmyd WX3D measurement. Additionally, the disc area and the rim volume are further parameters beside the CDR, displaying a negative mean difference (Table 2).

Comparison of CDR assessment with HRT III and Kowa nonmyd WX3D in the CDR ≥ 0.5 group showed a positive mean difference (0.06; limits of agreement −0.21 to 0.34). However, data from Kowa nonmyd WX3D exhibited the tendency to measure lower CDR values than HRT III in the group with CDR ≥ 0.5. The same tendency can be seen for the cup volume and the maximum cup depth; only the disc area and the rim volume showed a negative mean difference in the CDR ≥ 0.5 group (Table 2).

## 4. Discussion

This study evaluated the agreement of optic nerve head parameters obtained by Kowa nonmyd WX3D and HRT III with regard to the vertical CDR in glaucomatous patients for the first time. Comparison of optic nerve head parameters assessed with HRT III and Kowa nonmyd WX3D suggests a good agreement between those two devices indicated by the results of the Bland-Altman plots. Analysis showed the tendency to overestimate the CDR with Kowa nonmyd WX3D in relation to the HRT III measurements in the CDR < 0.5 group. CDR values measured with the Kowa nonmyd WX3D in the CDR ≥ 0.5 group were lower than HRT III CDR values, indicating the tendency to underestimate CDR with Kowa nonmyd WX3D in comparison to HRT III values in this group. These results are supported by earlier studies [18–20].

HRT III is an established tool for imaging and quantifying morphometric optic nerve head parameters. Optic nerve head assessment tools like OCT and GdX analysis do not include 3-dimensional optic disc evaluation. Furthermore, GdX data are of limited agreement with the HRT and OCT measurements can differ significantly between various OCT devices [21, 22]. In contrast to the HRT III, GdX, and OCT, the stereoscopic fundus images obtained by the Kowa nonmyd WX3D camera allow an additional evaluation of the optic disc regarding important in vivo parameters such as pallor, bleeding, and general appearance and can therefore serve as an additional assessment tool for optic nerve head vitality.

Optic nerve head imaging and especially HRT III have gained widespread use in monitoring optic nerve head parameters in patients with glaucoma or suspected glaucoma [23, 24]. Optic nerve head parameters assessed with HRT were demonstrated to be reproducible and help detect the onset and monitoring progression of glaucomatous optic neuropathy [10]. Test-retest variability has been extensively studied in the recent past and depends on patient age, severity of glaucoma, image quality, cylindrical error, lens opacity, surface geometry, and reference plane: in good-quality HRT images, the sensitivity for detecting 80% of rim area change of −0.012 mm^2^ per year requires, however, 4 examinations per year, which may not be feasible in everyday practice. Reducing the number of examinations to 2 per year reduces the sensitivity to 60% with simultaneous 20–30% false positive rate [25]. On the other hand stereographic photography is an accepted tool for optic disc assessment in glaucoma patients [26, 27]. Sensitivity and specificity for detecting progression of stereometric optic nerve head parameters with optic nerve head photography were analyzed in previous studies and seem to be limited: the best correlating optic nerve head parameter was the CDR [19, 28]. Reference height differences and the mean topography standard deviation (TSD) indicated image quality and were therefore influencing sensitivity and specificity of the optic nerve head assessment [29]. Further factors affecting the sensitivity and specificity including lens opacification and astigmatism have been found as factors affecting the measurement variability of this kind of optic disc tomography [28, 30, 31] and were therefore valuated as exclusion criteria.

The test-retest repeatability has been shown to improve with increasing CDR values (*R* ^2^ = 0.21; *p* < 0.01) in HRT measurements [32]. In this study test-retest repeatability was not investigated or compared. As only glaucoma patients were included, one could assume a rather large than a small CDR. However, our analyses divided the study population into two groups in accordance with the vertical CDR with a cut-off value of CDR 0.5. Thus differentiation between severity levels is solely based on a CDR classification and therefore limited. Furthermore functional aspects (like visual field parameters) are not included in this survey, so results from this study refer exclusively to morphometric but not functional criteria.

In this retrospective study both methods used a manual contour line alignment, which is a major source of measurement variability, even though simultaneous stereoscopic viewing of the optic nerve head photographs is known to facilitate the drawing of the contour line [24]. The contour line from the baseline measurement is normally automatically transferred to the follow-up measurement, which is also a common feature to reduce variability of CDR value comparison between a baseline and a follow-up measurement. The manually defined contour line affects the comparison of optic nerve head parameters in this study, due to visualized differences in optic disc parameters between HRT III and Kowa nonmyd WX3D (Figure 1), even though the mentioned difference in the disc area as a surrogate for the manually drawn contour line is within a small range between the two groups (Table 2). Recent HRT III software releases offer an automated analysis mode for optic nerve head assessment, which does not require prior manual outlining of a contour line [33]. Automated contour line drawing is still not commonly used, so manually selected outlining of disc boundaries was chosen in this study despite the above-mentioned variability. Nevertheless one should be cautious when interpreting transferred values between HRT III and Kowa nonmyd WX3D.

## 5. Conclusion

In conclusion, Kowa nonmyd WX3D is a potential tool for monitoring optic nerve head parameters quantitatively and qualitatively, not only as an adjunct or alternative to the HRT. Principle limitations of the study are the manual contour line alignment, the retrospective nature, and the very small sample sizes. However, Kowa nonmyd WX3D exhibited the tendency to measure larger CDR values than HRT III in the group with CDR < 0.5 and lower CDR values in the group with CDR ≥ 0.5.

## Figures and Tables

**Table 1:** Comparison and distribution of stereometric optic nerve head parameters assessed with HRT III and Kowa nonmyd WX3D for all investigated patients subdivided in two groups according to their CDR (cut-off value 0.5) and in total. Values are expressed as mean (± standard deviation, SD).

Optic nerve head parameter	HRT III (*N* = 24)	Kowa nonmyd WX3D (*N* = 16)
CDR < 0.5 group		
CDR	0.36 (±0.10)	0.50 (±0.12)
Disc area (mm^2^)	2.18 (±0.32)	2.83 (±0.57)
Cup volume (mm^3^)	0.18 (±0.10)	0.12 (±0.08)
Rim volume (mm^3^)	0.38 (±0.16)	0.50 (±0.35)
Maximum cup depth (mm)	0.70 (±0.19)	0.38 (±0.15)

CDR ≥ 0.5 group		
CDR	0.63 (±0.09)	0.48 (±0.12)
Disc area (mm^2^)	2.03 (±0.45)	2.79 (±0.59)
Cup volume (mm^3^)	0.30 (±0.16)	0.11 (±0.08)
Rim volume (mm^3^)	0.18 (±0.06)	0.51 (±0.32)
Maximum cup depth (mm)	0.72 (±0.25)	0.38 (±0.17)

Total		
CDR	0.46 (±0.17)	0.49 (±0.12)
Disc area (mm^2^)	2.12 (±0.38)	2.82 (±0.57)
Cup volume (mm^3^)	0.22 (±0.14)	0.11 (±0.08)
Rim volume (mm^3^)	0.30 (±0.16)	0.50 (±0.33)
Maximum cup depth (mm)	0.70 (±0.21)	0.38 (±0.15)

**Table 2:** Mean differences [limits of agreement (95% CI)] of stereometric optic nerve head parameters between HRT III and Kowa nonmyd WX3D assessment separated for the CDR < 0.5 group and the CDR ≥ 0.5 group and in total.

Optic nerve head parameter	CDR < 0.5 (*N* = 24)	CDR ≥ 0.5 (*N* = 16)	Total (*N* = 40)
CDR	−0.14 [−0.34 to 0.06]	0.06 [−0.21 to 0.34]	−0.06 [−0.36 to 0.24]
Disc area (mm^2^)	−0.66 [−1.73 to 0.42]	−0.76 [−1.92 to 0.40]	−0.70 [−1.80 to 0.40]
Cup volume (mm^3^)	0.06 [−0.11 to 0.23]	0.19 [−0.07 to 0.46]	0.11 [−0.13 to 0.36]
Rim volume (mm^3^)	−0.12 [−0.87 to 0.63]	−0.32 [−0.95 to 0.28]	−0.20 [−0.93 to 0.53]
Maximum cup depth (mm)	0.32 [−0.04 to 0.68]	0.34 [−0.01 to 0.69]	0.33 [−0.02 to 0.68]
